# Cell-free DNA copy number variations predict efficacy of immune checkpoint inhibitor-based therapy in hepatobiliary cancers

**DOI:** 10.1136/jitc-2020-001942

**Published:** 2021-05-10

**Authors:** Xu Yang, Ying Hu, Keyan Yang, Dongxu Wang, Jianzhen Lin, Junyu Long, Fucun Xie, Jinzhu Mao, Jin Bian, Mei Guan, Jie Pan, Li Huo, Ke Hu, Xiaobo Yang, Yilei Mao, Xinting Sang, Jiao Zhang, Xi Wang, Henghui Zhang, Haitao Zhao

**Affiliations:** 1 Department of Liver Surgery, State Key Laboratory of Complex Severe and Rare Diseases, Peking Union Medical College Hospital, Chinese Academy of Medical Sciences and Peking Union Medical College, Beijing, China; 2 Center of Integrative Medicine, Beijing Ditan Hospital, Capital Medical University, Beijing, China; 3 Genecast Biotechnology Co., Ltd, Wuxi, China; 4 Department of Medical Oncology, Peking Union Medical College Hospital, Chinese Academy of Medical Sciences and Peking Union Medical College, Beijing, China; 5 Department of Radiology, Peking Union Medical College Hospital, Chinese Academy of Medical Sciences and Peking Union Medical College, Beijing, China; 6 Department of Nuclear Medicine, Peking Union Medical College Hospital, Chinese Academy of Medical Sciences and Peking Union Medical College, Beijing, China; 7 Center of Radiotherapy, Peking Union Medical College Hospital, Chinese Academy of Medical Sciences and Peking Union Medical College, Beijing, China; 8 Department of Immunology, School of Basic Medical Sciences, Capital Medical University, Beijing, China; 9 Beijing Shijitan Hospital, Capital Medical University, Beijing, China; Ninth School of Clinical Medicine, Peking University, Beijing, China; School of Oncology, Capital Medical University, Beijing, China; 10 Institute of Infectious Diseases, Beijing Ditan Hospital, Capital Medical University, Beijing, China

**Keywords:** immunotherapy, liver neoplasms, tumor biomarkers

## Abstract

**Background:**

This study was designed to screen potential biomarkers in plasma cell-free DNA (cfDNA) for predicting the clinical outcome of immune checkpoint inhibitor (ICI)-based therapy in advanced hepatobiliary cancers.

**Methods:**

Three cohorts including 187 patients with hepatobiliary cancers were recruited from clinical trials at the Peking Union Medical College Hospital. Forty-three patients received combination therapy of programmed cell death protein 1 (PD-1) inhibitor with lenvatinib (ICI cohort 1), 108 patients received ICI-based therapy (ICI cohort 2) and 36 patients received non-ICI therapy (non-ICI cohort). The plasma cfDNA and blood cell DNA mutation profiles were assessed to identify efficacy biomarkers by a cancer gene-targeted next-generation sequencing panel.

**Results:**

Based on the copy number variations (CNVs) in plasma cfDNA, the CNV risk score model was constructed to predict survival by using the least absolute shrinkage and selection operator Cox regression methods. The results of the two independent ICI-based therapy cohorts showed that patients with lower CNV risk scores had longer overall survival (OS) and progression-free survival (PFS) than those with high CNV risk scores (log-rank p<0.01). In the non-ICI cohort, the CNV risk score was not associated with PFS or OS. Furthermore, the results indicated that 53% of patients with low CNV risk scores achieved durable clinical benefit; in contrast, 88% of patients with high CNV risk scores could not benefit from combination therapy (p<0.05).

**Conclusions:**

The CNVs in plasma cfDNA could predict the clinical outcome of the combination therapy of PD-1 inhibitor with lenvatinib and other ICI-based therapies in hepatobiliary cancers.

## Introduction

Hepatobiliary cancers include a spectrum of lethal carcinomas arising in the liver (hepatocellular carcinoma (HCC)), biliary tract (intrahepatic and extrahepatic cholangiocarcinoma) and gallbladder (gallbladder cancer (GBC)), and many patients are diagnosed with advanced disease.^1 2^ Immune checkpoint inhibitor (ICI) therapy or targeted therapy monotherapy has shown considerable efficacy in advanced hepatobiliary cancers.^3–9^ Moreover, clinical trials have shown that the combination of ICI therapy with targeted therapy has encouraging efficacy in hepatobiliary cancers.^10–13^ The IMbrave150 trial, a phase III randomized study, showed that atezolizumab combined with bevacizumab resulted in better overall survival (OS) and progression-free survival (PFS) than sorafenib in patients with unresectable HCC.^10^ Recently, a phase Ib study found that lenvatinib plus a PD-1 inhibitor (pembrolizumab or nivolumab) had a promising objective response rate (ORR) and PFS for unresectable HCC.^11 14^ Our previous study indicated that treatment with lenvatinib plus a PD-1 inhibitor is an effective and safe strategy in patients with advanced biliary tract cancer (BTC).^12 13^

However, only a subset of patients benefit from ICI-based therapy. There are no definitive biomarkers for predicting the response to the combination of ICI therapy with targeted therapy in hepatobiliary cancers. Several studies have shown trends toward patients with HCC with positive programmed death-ligand 1 (PD-L1) expression having a higher response to immune checkpoint inhibition monotherapy.^3 15^ For BTCs treated with nivolumab, PD-L1 expression was also associated with better PFS and OS.^5^ In clinical practice, biomarkers based on tumor tissue have some shortcomings, including the difficulty of obtaining enough tumor tissue as well as spatial heterogeneity. Clinical studies have indicated that cell-free DNA (cfDNA), as a non-invasive tool, provides a promising method for the screening and diagnosis of cancer, the prediction of the response to therapy, the early diagnosis of relapse and the detection of secondary resistance.^16–18^ Moreover, real-time liquid biopsy biomarkers are also expected to assist in clinical decision making, including patient selection and the prediction of immunotherapy efficacy.^19–22^

This study was designed to screen potential biomarkers in plasma cfDNA for predicting the efficacy of the combination therapy of PD-1 inhibitor with lenvatinib and ICI-based therapy in hepatobiliary cancers.

## Methods

### Patients

**Figure 1:**
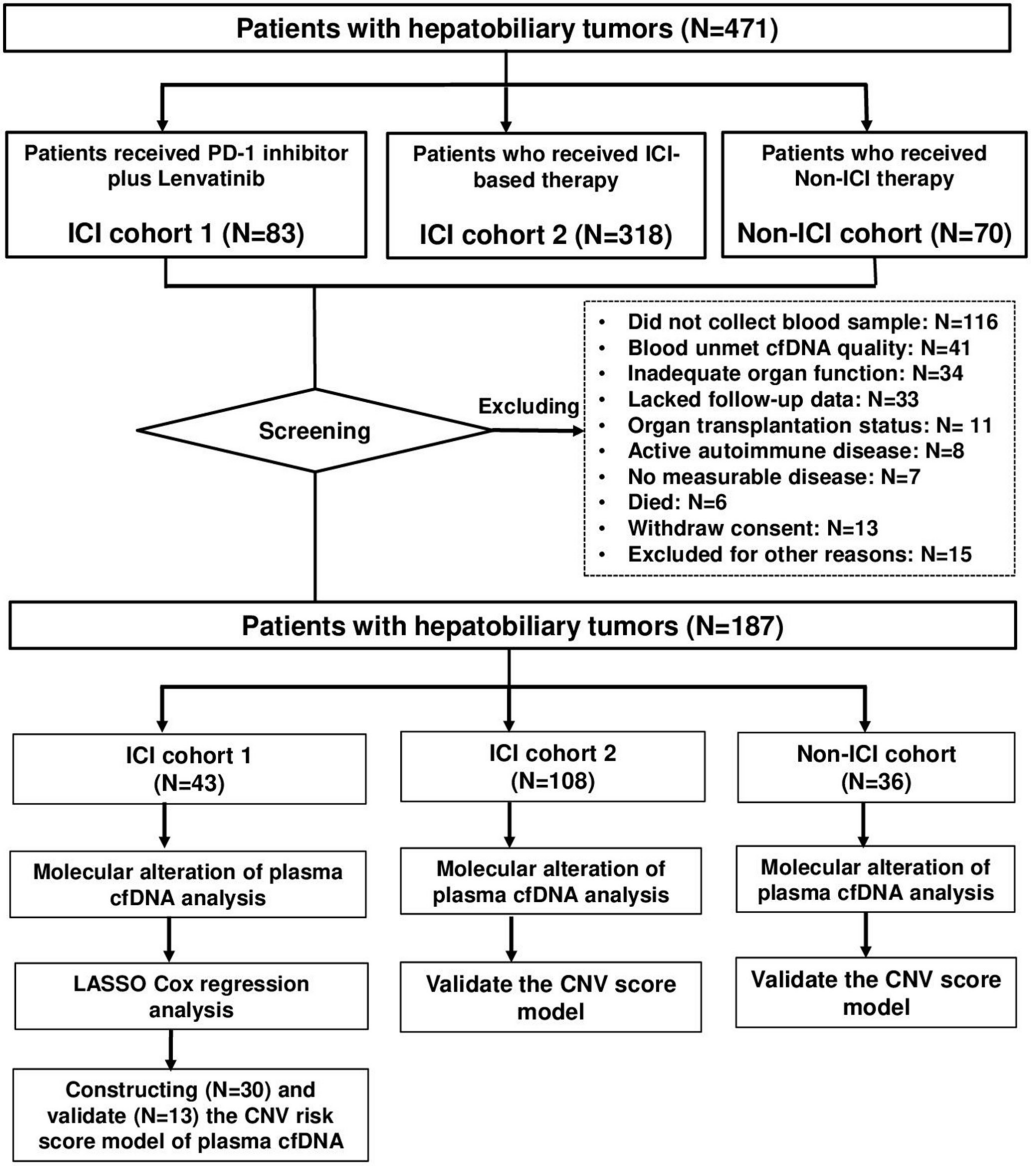
Flow chart of the study design and the data selection process. A total of 471 patients with hepatobiliary cancers were screened. After excluding 284 patients with reasons, three cohorts including 187 patients with hepatobiliary cancers were recruited from clinical trials at the Peking Union Medical College Hospital. Forty-three patients received combination therapy of PD-1 inhibitor with lenvatinib (ICI cohort 1), 108 patients received ICI therapy (ICI cohort 2) and 36 patients received non-ICI therapy (non-ICI cohort). cfDNA, cell-free DNA; CNV, copy number variation; ICI, immune checkpoint inhibitor; LASSO, least absolute shrinkage and selection operator; PD-1, programmed cell death protein 1.

A total of 471 patients with hepatobiliary cancers were screened from two clinical trials (NCT03895970, NCT03892577) at the Peking Union Medical College Hospital. Eligible patients with hepatobiliary cancers who met the inclusion and criteria were recruited to the present study. The main inclusion criteria for patients with hepatobiliary cancer were as follows: (1) pathologically confirmed as hepatobiliary cancer or confirmed by imaging as HCC (by the American Association for the Study of Liver Diseases or standard for the diagnosis and treatment of primary liver cancer 2017 in China)^23 24^; (2) Eastern Cooperative Oncology Group performance status (ECOG PS) score 0–2 and (3) life expectancy of at least 3 months. Two hundred eighty-four patients were excluded based on the following criteria: did not collect blood sample (n=116), blood unmet cfDNA quality (n=41), inadequate organ function (n=34), lacked follow-up data (n=33), organ transplantation status (n=11), active autoimmune disease (n=8), no measurable disease (n=7), died (n=6), withdrew consent (n=13) and excluded for other reasons (n=15). A total of 187 patients with hepatobiliary cancers were divided into three cohorts according to therapy. ICI cohort 1 consisted of 43 patients with hepatobiliary cancers who received combination therapy of PD-1 inhibitor with lenvatinib. ICI cohort 2 included 108 patients with hepatobiliary cancers who received ICI-based therapy. The non-ICI cohort included 36 patients with hepatobiliary cancers who received non-ICI therapy (figure 1).

### Assessment of clinical outcomes

The objective response was measured according to the Response Evaluation Criteria in Solid Tumors V.1.1 guidelines,^25^ and durable clinical benefit (DCB) was defined as complete response (CR), partial response (PR) or stable disease (SD) for ≥24 weeks,^26^ which were evaluated by professional radiologists at our center who were blinded to the therapeutic outcomes and clinicopathological features. For cohort 1 and cohort 2, PFS was defined as the time from the start of anti-PD-1/PD-L1 treatment to the first documented disease progression or death from any cause. OS was defined as the time between the start of anti-PD-1/PD-L1 treatment and death due to any cause. For non-ICI cohort 3, PFS and OS were evaluated as the time from the start of the systematic therapy.

### DNA extraction, next-generation sequencing and genomic feature analysis of cfDNA

The plasma cfDNA and blood cell DNA mutation profiles were assessed by a cancer gene-targeted next-generation sequencing (NGS) panel. Methods of DNA extraction, target capture and NGS are provided in the online supplemental material. Algorithm of genomic feature including tumor mutation burden (TMB), copy number instability (CNI) and molecular mutation burden (MMB) are provided in the online supplemental material.

10.1136/jitc-2020-001942.supp1 Supplementary data

### Tumor burden score

The tumor burden score (TBS) was calculated by the maximum tumor size and number of tumors^27 28^ using the following formula:

$$TBS^{2}=Maximuntumordiameter^{2}+Numberoftumors^{2}$$

### Construction of a prognostic model

A total of 43 patients were randomly allocated to the discovery set (n=30) and validation set (n=13). Univariate Cox regression analysis was performed to analyze the gene copy number value significantly associated with OS. Least absolute shrinkage and selection operator (LASSO) Cox regression analysis was used to determine the coefficient for each feature and estimate the likelihood deviance. The coefficients and partial likelihood deviance were calculated by the ‘glmnet’ package in R. The copy number variation (CNV) risk score was calculated by the following formula:

$$CNVriskscore=\sum_{i=1}^{n}Coef_{i}*x_{i}$$

where *Coef_i_* is the risk coefficient of each factor calculated by the LASSO Cox model, and *x_i_* is the copy number value of each factor.

The time-dependent receiver operating characteristic (tROC) curve to detect the predictive power of the risk score was constructed by the ‘survival ROC’ package, and Kaplan-Meier analysis with the log-rank test was carried out by the ‘survival’ package in R. The optimal cut-off value of the CNV risk score was determined by the Youden Index.

### Statistical analysis

The clinicopathological features of the discovery and validation cohorts were compared using the χ^2^ test (two-tailed). OS and PFS were analyzed by multivariate Cox regression and the Kaplan-Meier method, and the log-rank test was used to detect the significant differences between different groups via the ‘survival’ and ‘survminer’ packages in R. The data are expressed as the median and IQR. The Wilcoxon rank-sum test was applied to assess the differences between two groups. Fisher’s exact test was used to analyze the correction between CNV risk score with clinicopathological characteristics. P<0.05 was considered significant for two-sided tests. Statistical analyses were performed using R V.3.6.2.

## Results

### Clinical characteristics of the study cohorts

The detailed clinical characteristics are shown in table 1 and online supplemental table S1. Four hundred seventy-one patients with hepatobiliary cancers were screened, and 284 patients were excluded. Then, a total of 187 patients with hepatobiliary cancers were divided into three cohorts according to therapy. ICI cohort 1 consisted of 43 patients with hepatobiliary cancers who received combination therapy of PD-1 inhibitor with lenvatinib. ICI cohort 1 included 12 patients with HCC, 19 patients with intrahepatic cholangiocarcinoma (ICC), 4 patients with extrahepatic cholangiocarcinoma (ECC), 5 patients with GBC and 3 patients with combined hepatocellular-cholangiocarcinoma (CHCC). The median follow-up was 7.87 months, and 30.2% of 43 patients reached DCB from PD-1 inhibitor plus lenvatinib therapy during the follow-up period. ICI cohort 2 included 108 patients with hepatobiliary cancers who received ICI therapy. ICI cohort 2 included 44 patients with HCC, 39 patients with ICC, 16 patients with ECC, 8 patients with GBC and 1 patient with CHCC. The median follow-up was 11.05 months, and 50% of 108 patients reached DCB from ICI therapy. In the non-ICI cohort, the median follow-up was 5.83 months, and 30.6% of 36 patients reached DCB. Sex, histological type and histological grade were comparable among the three cohorts (p>0.05).

10.1136/jitc-2020-001942.supp2 Supplementary data

**Table 1:** Key clinical characteristics of patients with hepatobiliary cancer (n=187)

Variable	ICI cohort 1 (n=43)	ICI cohort 2 (n=108)	Non-ICI cohort (n=36)
Median age (range)	61(27–82)	59.5 (18–80)	61.5 (34–84)
Sex—no. (%)			
Male	30 (69.8)	68 (63.0)	26 (72.2)
Female	13 (30.2)	40 (37.0)	10 (27.8)
Histological type—no. (%)			
HCC	12 (27.9)	44 (40.7)	11 (30.6)
ICC	19 (44.2)	39 (36.1)	11 (30.6)
ECC	4 (9.3)	16 (14.8)	7 (19.4)
GBC	5 (11.6)	8 (7.4)	6 (16.7)
CHCC	3 (7.0)	1 (0.9)	1 (2.8)
ECOG PS—no. (%)			
0	25 (58.1)	40 (37.0)	12 (33.3)
1	12 (27.9)	60 (55.6)	18 (50)
2	6 (14.1)	8 (7.4)	6 (16.7)
Child-Pugh grade—no. (%)			
A	38 (88.4)	95 (88.0)	29 (80.6)
B	5 (11.6)	11 (10.2)	5 (13.9)
C	0 (0.0)	2 (1.9)	2 (5.6)
Tumor burden score—no. (%)			
≥8	15 (34.9)	42 (38.9)	10 (27.8)
<8	28 (65.1)	66 (61.1)	26 (72.2)
Number of prior systemic therapies for advanced metastatic disease—no. (%)			
0	28 (65.1)	44 (40.7)	24 (66.7)
1	12 (27.9)	39 (36.1)	4 (11.1)
2 and more	3 (7.0)	25 (23.1)	8 (22.2)
Current therapy—no. (%)			
De novo combination PD-1 inhibitor with lenvatinib	43 (100)	32 (29.6)	0 (0.0)
Lenvatinib sequential PD-1 combination therapy	0 (0.0)	27 (25.0)	0 (0.0)
PD-1 inhibitor+other target therapy	0 (0.0)	49 (45.4)*	0 (0.0)
Target therapy monotherapy	0 (0.0)	0 (0.0)	26 (72.2)†
Others	0 (0.0)	0 (0.0)	10 (27.8)
Follow-up—(IQR) month	7.87 (5.8–11.3)	11.05 (6.68–14.83)	5.83 (3.44–8.26)

*Consists of apatinib (n=23), bevacizumab (n=5) and anlotinib (n=2), regorafenib (n=2), cabozantinib (n=2) and others.

†Lenvatinib (n=16), afatinib (n=2), olaparib (n=2) and others.

CHCC, combined hepatocellular-cholangiocarcinoma; ECC, extrahepatic cholangiocarcinoma; GBC, gallbladder cancer; HCC, hepatocellular carcinoma; ICC, intrahepatic cholangiocarcinoma; ICI, immune checkpoint inhibitor; PD-1, programmed cell death protein 1; PS, performance status.

### Construction and validation of the plasma cfDNA CNV risk score to predict survival after combination therapy

Samples from 187 patients with hepatobiliary cancers were collected. Plasma cfDNA and white blood cell DNA were sequenced by a cancer gene-targeted NGS panel. The mutation profiles of 10 canonical pathways, including the cell cycle, Hippo, Myc, Notch, Nrf2, PI3 kinase/Akt, RTK-RAS, TGFβ signaling, p53 and β-catenin/WNT pathways, were assessed.^29^ The *TP53, RICTOR, NF1, CDKN2A, RB1, FBXW7, NFE2L2, PIK3CA, STK11, ERBB4, KRAS* and *NRAS* genes were frequently mutated in hepatobiliary cancers pretreatment (online supplemental figure S1). The landscape of the CNVs in plasma cfDNA is shown in online supplemental figure S2.

10.1136/jitc-2020-001942.supp3 Supplementary data

10.1136/jitc-2020-001942.supp4 Supplementary data

Next, we assessed whether the CNVs in plasma cfDNA were predictive of the response to combination therapy of PD-1 inhibitor with lenvatinib. Forty-three patients were randomly divided into two groups: the discovery cohort (HCC=7, ICC=13, ECC=3 and others=7) and the validation cohort (HCC=5, ICC=6, ECC=1 and GBC=1). Using LASSO Cox regression methods, the genes with CNVs in plasma cfDNA were selected to build the CNV score model to predict survival after combination therapy in the discovery cohort. The formula for the CNV score was based on the copy number of eight genes, including *CALR*, *NR4A3*, *IDH2*, *IGF1R*, *ETV6*, *STAT3*, *NF2* and *CTCF*. The formula for the CNV score was as follows: CNV risk score=(−1.4267831)*×CALR* +0.5515164*×STAT3*+1.5124620*×IDH2*+1.5372432*×ETV6*+4.1835445*×IGF1R*+(−1.3164812)*×NR4A3*+1.1780366*×NF2*+1.5359307*×CTCF* (online supplemental figure S3).

10.1136/jitc-2020-001942.supp5 Supplementary data

**Figure 2:**
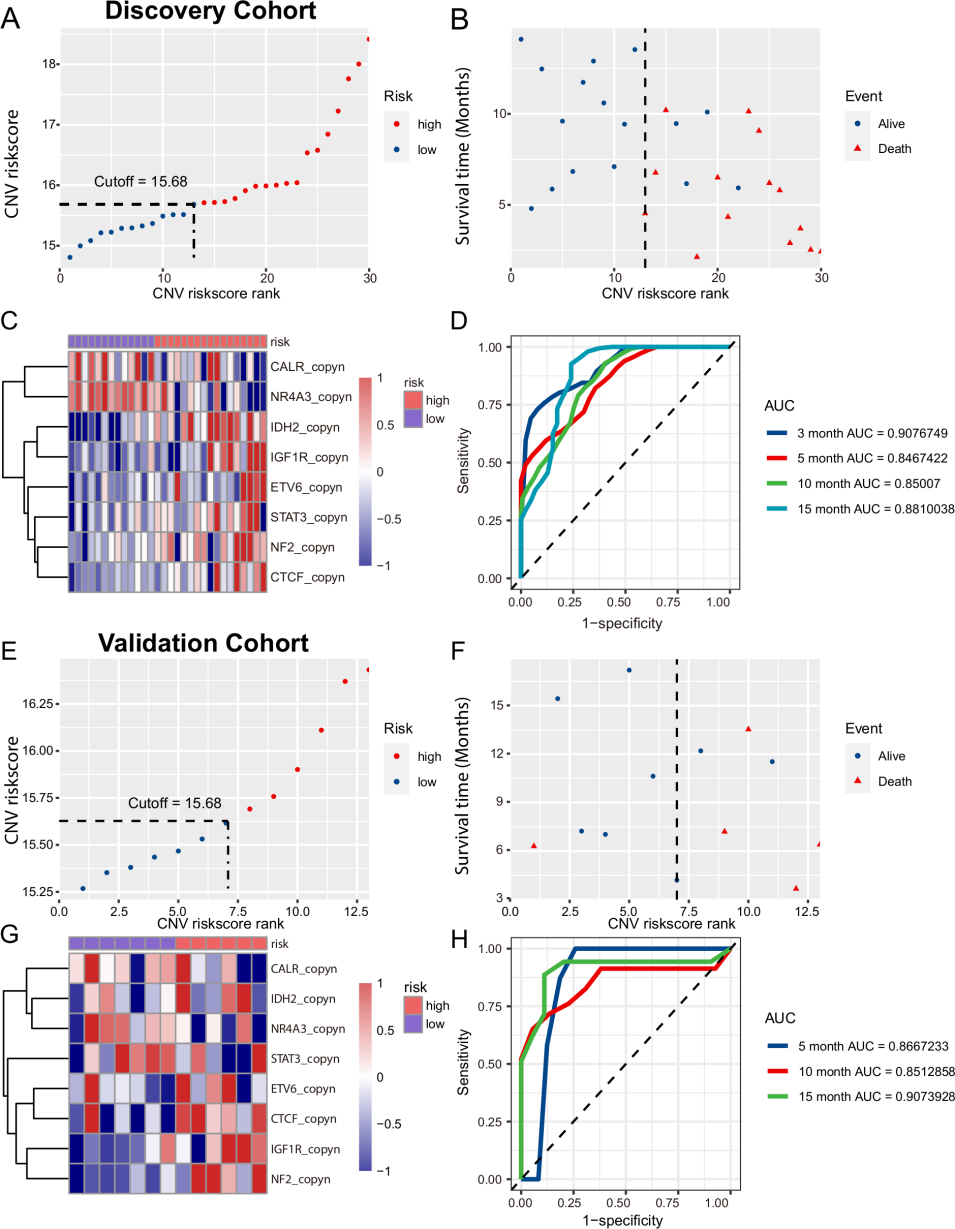
Construction and assessment of the copy number variation (CNV) risk score for hepatobiliary cancers. (A, E) The CNV risk scores of the patients in the discovery and validation cohorts sorted in ascending order. (B, F) Distributions of vital status for each patient according to the CNV risk score levels. (C, G) The copy number Z-scores and level of CNV risk scores of *CALR*, *NR4A3*, *IDH2*, *IGF1R*, *ETV6*, *STAT3*, *NF2* and *CTCF* are shown in the heatmap. (D, H) The area under the curve (AUC) of the time-dependent receiver operating characteristic (ROC) curve was 0.881 in the discovery cohort and 0.907 in the validation cohort for the CNV risk score.

**Figure 3:**
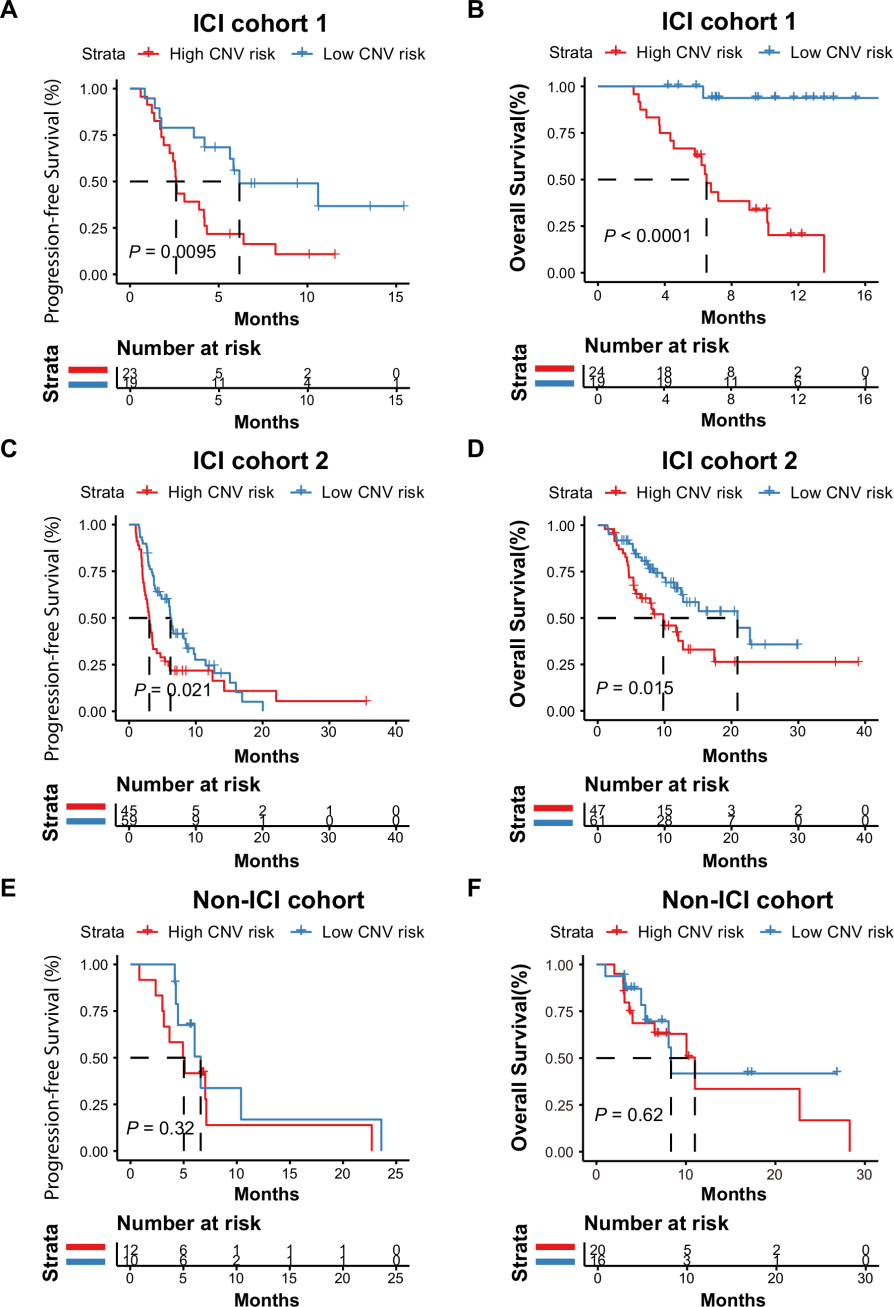
Association between the plasma cell-free DNA (cfDNA) copy number variation (CNV) risk score and the response to immune checkpoint inhibitor (ICI) therapy. (A and B) Kaplan-Meier curves for the overall survival (OS) and progression-free survival (PFS) of patients with hepatobiliary cancer in ICI cohort 1 stratified into high CNV risk and low CNV risk score groups. The PFS log-rank test showed p=0.0095; low versus high CNV risk, median PFS: 6.17 months vs 2.60 months, HR=0.045. The OS log-rank test showed p<0.0001; low vs high CNV risk, median OS: not reached vs 6.5 months, HR=0.39. (C and D) Kaplan-Meier curves for the PFS and OS of patients with hepatobiliary cancer in ICI cohort 2 stratified into high CNV risk and low CNV risk score groups. The log-rank test of PFS in different CNV risk score groups showed p=0.021; low versus high CNV risk score: median PFS, 6.2 months vs 3.033 months, HR=0.61. The OS log-rank test showed p=0.015; low vs high CNV risk score: median OS, 20.9 months vs 9.8 months, HR=0.50. (E and F) Kaplan-Meier curves for the PFS and OS of patients with hepatobiliary cancer in the non-ICI cohort stratified into high CNV risk and low CNV risk score groups. The PFS log-rank test showed p=0.32; low versus high CNV risk, median PFS: 6.60 months vs 5.02 months. The OS log-rank test showed p=0.62, low versus high CNV risk, median OS: 8.33 months vs 1.00 months.

**Figure 4:**
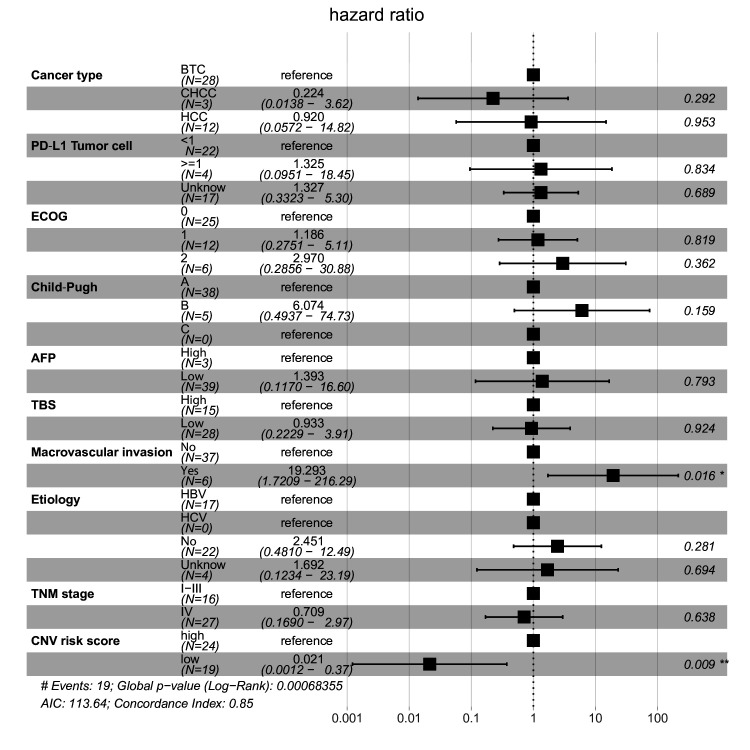
Effect of the copy number variation (CNV) risk score on overall survival after combination therapy of programmed cell death protein 1 (PD-1) inhibitor with lenvatinib, by clinical characteristics. Forest plot for overall survival in subgroups. Estimates are based on a Cox proportional hazards model. AFP, alpha-fetoprotein; ECOG, Eastern Cooperative Oncology Group; PD-L1, programmed death-ligand 1; TBS, tumor burden score; TNM, tumor, node, metastases.

To validate the CNV risk score of plasma cfDNA to predict survival after combination therapy, R packages ‘survminer’ were used to generate the optimum cut-off value of the CNV score. In the training cohort, we included 30 patients with a CNV risk score of baseline plasma cfDNA higher than 15.68 (high-risk group) with shorter survival times after combination therapy of PD-1 inhibitor with lenvatinib and those with a CNV risk score of baseline plasma cfDNA lower than 15.68 (low-risk group) with longer survival times after combination therapy (figures 2A, C and 3B). We analyzed the CNV risk score and OS of 13 patients from cohort 1 that were not used as part of the training set. The result showed that the patients with high CNV risk score had median OS of 10.37 months and patients with low CNV risk score did not reach (p=0.11) (online supplemental figure 5A). In addition, we compared the CNV risk score between the DCB and no durable benefit (NDB) groups and the CNV risk score among the PR, PD and SD groups, and the results showed the CNV risk score did not significantly change among those groups of 13 patients (online supplemental figure 5B, C). In the validation cohort, the optimum cut-off of the CNV score was the same as that in the discovery cohort, and the results were similar to those in the discovery cohort (figure 2E, F, G). When the distribution of the CNV risk score and survival status were assessed in the training and validation cohorts, the results showed that patients with lower CNV risk scores had better survival than those with higher CNV risk scores (figure 2B, F). The tROC curves of the CNV risk score in the discovery and validation cohorts are shown in figure 4. In the discovery cohort, the CNV risk score had an area under the curve (AUC) of 0.908 at 3 months, 0.847 at 5 months, 0.850 at 10 months and 0.881 at 15 months (figure 2D). In the validation cohort, the CNV risk score had an AUC of 0.867 at 5 months, 0.851 at 10 months and 0.907 at 15 months (figure 2H).

10.1136/jitc-2020-001942.supp6 Supplementary data

In ICI cohort 1, Kaplan-Meier curves showed that all patients with hepatobiliary cancer with low CNV risk scores (n=20) had longer OS and PFS than patients with hepatobiliary cancer with high CNV risk scores (n=23) (figure 3A, low vs high CNV risk score, PFS: HR=0.39, log-rank p=0.0095; figure 3B, OS: HR=0.045, log-rank p<0.0001). During the follow-up period, the median OS of patients with hepatobiliary cancer with low CNV risk scores was not reached, and the median OS of patients with hepatobiliary cancer with high CNV risk scores was 6.5 months. The median PFS of patients with hepatobiliary cancer with low and high CNV risk scores was 6.17 months and 2.60 months, respectively.

To validate this finding, we further analyzed the 108 patients with hepatobiliary cancer from independent ICI cohort 2 from our clinical trials. The results were similar to those of ICI cohort 1. Favorable OS and PFS were observed in patients with hepatobiliary cancer with low CNV risk scores (figure 3C, low vs high CNV risk score: median PFS, 6.2 months vs 3.03 months, HR=0.61, PFS log-rank p=0.021; figure 3D, median OS, 20.90 months vs 9.80 months, HR=0.50, OS log-rank p=0.015). In the non-ICI cohort, OS and PFS were not significantly different between patients with low and high CNV risk scores (figure 3E, F, PFS log-rank p=0.32, OS log-rank p=0.62).

To further confirm the effect of the CNV risk score of plasma cfDNA at baseline on the OS of patients with hepatobiliary cancer who received combination therapy, multiple Cox regression was used to assess the effect of multiple factors, including cancer type, PD-L1 expression, ECOG score, Child-Pugh score, alpha-fetoprotein (AFP), TBS, macrovascular invasion, etiology, TNM stage and CNV risk score, on OS. The results showed that the CNV risk score of plasma cfDNA was an independent factor for predicting the OS of patients with hepatobiliary cancer who received combination therapy of PD-1 inhibitor with lenvatinib (figure 4, global log-rank p=0.0068; C-index=0.85; CNV risk score: HR=0.021, p=0.009). Using Fisher’s exact test in ICI cohorts 1 and 2, we found that the CNV risk score (high/low) was associated with histological type (online supplemental table S2, p<0.05), TBS (online supplemental table S2, p<0.05) and maximum tumor diameter (online supplemental table S2, p<0.05), but not with sex, histological grade or macrovascular invasion (online supplemental table S2, p>0.05).

10.1136/jitc-2020-001942.supp7 Supplementary data

### Association between the plasma cfDNA CNV risk score and the response to ICI therapy

**Figure 5:**
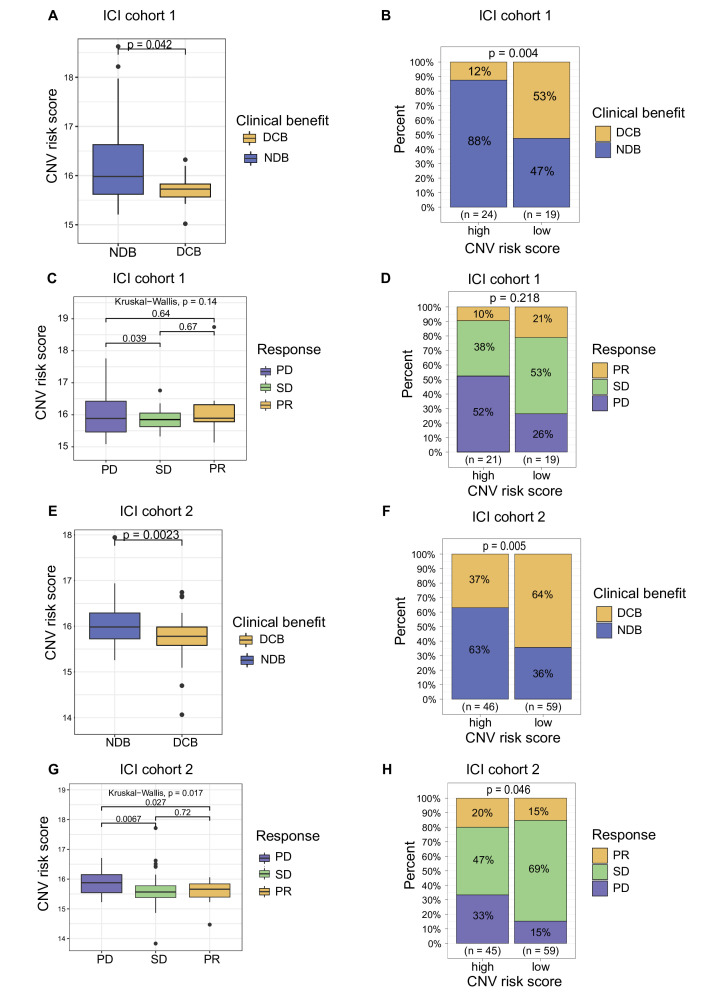
Association between the copy number variation (CNV) risk score of plasma cell-free DNA (cfDNA) and the response to immune checkpoint inhibitor (ICI)-based therapy. (A) CNV risk score levels of patients in the durable clinical benefit (DCB) and no durable benefit (NDB) groups from ICI cohort 1. (B) Proportional representation of DCB and NDB in ICI cohort 1 based on the level of the CNV risk score. (C) The CNV risk score levels of patients in the partial response (PR), stable disease (SD) and progressive disease (PD) groups from ICI cohort 1. (D) Proportional representation of the objective response rate (ORR) in ICI cohort 1 based on level of the CNV risk score. (E) CNV risk score levels of patients in the DCB and NDB groups from ICI cohort 2. (F) Proportional representation of DCB and NDB in ICI cohort 2 based on the level of the CNV risk score. (G) CNV risk score levels of patients in the PR, SD and PD groups from ICI cohort 2. (H) Proportional representation of the ORR in ICI cohort 2 based on the level of the CNV risk score.

In ICI cohort 1, the CNV risk score of plasma cfDNA was lower in patients showing DCB than in those showing NDB (figure 5A, p=0.042). When the optimum cut-off of the CNV score was used, the result from ICI cohort 1 showed that 53% of patients in the low CNV risk group achieved DCB after combination therapy of PD-1 inhibitor with lenvatinib. In contrast, 12% of patients in the high CNV risk group achieved DCB, and 88% of patients could not benefit from combination therapy of PD-1 inhibitor with lenvatinib (figure 5B, DCB: 53% vs 12%, NDB: 47% vs 88%, p=0.004). In ICI cohort 1, there was a lower tendency of PD and a higher tendency of SD and PR in the low CNV risk group than in the high CNV risk group (figure 5D, PD: 26% vs 52%, SD: 53% vs 38%, PR: 21% vs 10%, p=0.218; disease control rate (DCR), 74% vs 48%, p=0.093). Similar results were observed in ICI cohort 2. The CNV risk score of plasma cfDNA was lower in patients showing DCB than in those showing NDB (figure 5E, p=0.0023). Compared with that in the high CNV risk group, a higher percentage of patients in the low CNV risk group achieved DCB after ICI therapy (figure 5F; DCB: 64% vs 37%, NDB: 36% vs 63%, p=0.005). The CNV risk score was higher in the PD group than in the SD and PR groups in both ICI cohort 1 and ICI cohort 2 (figure 5C, G; ICI cohort 1: PD vs SD, p=0.039, PD vs PR, not significant; ICI cohort 2: PD vs SD, p=0.0067, PD vs PR, p=0.027). In ICI cohort 2, the percentage of PD was significantly lower and the percentages of SD and PR were significantly higher in the low CNV risk group than in the high CNV risk group (figure 5H, PR: 15% vs 20%, SD: 69% vs 47%, PD: 15% vs 33%, p=0.046; DCR: 85% vs 63%, p=0.011).

The molecular features of hepatobiliary cancers with high and low CNV risk scores were investigated. Hepatobiliary cancers with high CNV risk scores were characterized by high TMB and MMB. Moreover, higher CNI scores were observed in hepatobiliary cancers with high CNV risk scores than in hepatobiliary cancers with low CNV risk scores (online supplemental figure S4, p<0.05).

10.1136/jitc-2020-001942.supp8 Supplementary data

## Discussion

ICI-based therapy may provide new clinical strategies for patients with hepatobiliary cancers. This study constructed a CNV risk score model of plasma cfDNA to predict the clinical outcome of the combination therapy of PD-1 inhibitor with lenvatinib and other ICI-based therapies in hepatobiliary cancers.

Chromosomal instability is a hallmark of cancer biology.^30^ Several studies have indicated that CNVs identified in tumor tissue is related to the response to immunotherapy in patients with cancer.^31–34^ A recent study found that HCC tumors with a low burden of broad copy number chromosomal alterations display higher immune infiltration and have a better response rate to anti-PD-1 inhibitors than those with a median/high broad copy number.^35^ Moreover, Davoli *et al*^31^ found that a higher CNV burden in tumor tissue correlated with immune escape and poorer survival in patients with metastatic melanoma treated with immunotherapy. Regarding liquid biopsy, Weiss *et al*^36^ constructed a CNI scoring system based on plasma cfDNA and found that the CNI score could be used as an early indicator of the response to immunotherapy for diverse advanced cancers. Another study also found that the cfDNA genome instability number (GIN) could discriminate atypical responses (such as pseudoprogression or hyperprogressive disease) and could monitor the response to ICI therapy.^37^ However, the association between CNV and immunotherapy in hepatobiliary cancer is still unclear. In this study, by using LASSO Cox regression, we constructed a CNV risk score model to predict the response to combination therapy. The results of the tROC analysis were encouraging, and the CNV risk score had an AUC >0.8. In the PD-1 inhibitor and lenvatinib combination therapy cohort, favorable OS and PFS were observed in patients with hepatobiliary cancer with low CNV risk scores. These results were confirmed in another independent ICI-based cohort of patients with hepatobiliary cancer. These findings are based on pretreatment plasma cfDNA alterations, which could provide new biomarkers and information for clinicians to make appropriate clinical decisions for each patient. In the non-ICI cohort, the CNV risk score was not associated with the clinical outcome of patients. Our findings indicated that the patients with hepatobiliary cancers with low cfDNA CNV risk score benefit from the PD-1 inhibitor with lenvatinib and other ICI-based therapies. In addition, we further investigated the molecular features of tumors with low and high CNV risk scores, and the results showed that tumors with high CNV risk scores were characterized as highly malignant, as reflected by the TMB, MMB and CNI score. A recent study reported that HCC with high broad CNV score showed high mutational burdens,^35^ which was similar to our study. In addition, this study indicated that patients with HCC with high broad CNV scores had lower ratio of observed/expected neo-antigens. It suggested that the accumulation of CNV may be associated with an enhanced editing of non-antigenic mutations, regardless of overall mutation burdens.^35^ These findings may explain the poor clinical outcome of patients with tumors with high CNV risk scores treated with ICI-based therapy.

Although ICI-based therapy has led to evolutionary developments in cancer management, some patients still do not respond to therapy. In our PD-1 inhibitor and lenvatinib combination therapy cohort, 30.2% of patients achieved DCB from combination therapy, and 69.8% of patients could not benefit from the combination therapy. In clinical practice, the main concern is selecting patients who would benefit from the therapy. Meanwhile, excluding patients who would not benefit from therapy is also equally important. In our study, we found that >50% of patients with low CNV risk scores achieved DCB from combination therapy. In contrast, only 12% of patients with high CNV risk scores achieved DCB from combination therapy, and 88% of patients with high CNV risk scores could not benefit from combination therapy. Our findings suggested that patients with low CNV risk scores were more likely to benefit from combination therapy; in contrast, patients with high CNV risk scores were less likely to benefit from combination therapy.

There were several limitations in the present study. This is a single-center retrospective study and the cohorts were relatively heterogeneous among cancer types or ICI-based regimens. Moreover, this CNV risk score model was constructed based on a small number of patients, therefore, further larger independent studies and multicenter should be designed to validate the clinical value of plasma cfDNA CNVs in predicting immunotherapy efficacy in hepatobiliary cancers.

## Conclusions

In summary, the present study constructed a CNV risk score model based on plasma cfDNA to predict survival after combination therapy of PD-1 inhibitor with lenvatinib and other ICI-based therapies in hepatobiliary cancers. In clinical practice, the cfDNA CNV risk score may provide valuable resources for personalized hepatobiliary cancer combination immunotherapy regimens.

10.1136/jitc-2020-001942.supp9 Supplementary data

10.1136/jitc-2020-001942.supp10 Supplementary data

10.1136/jitc-2020-001942.supp11 Supplementary data

10.1136/jitc-2020-001942.supp12 Supplementary data

## Data Availability

Data are available on reasonable request.
