# Supplementary material for: Cell-free DNA copy number variations predict efficacy of immune checkpoint inhibitor-based therapy in hepatobiliary cancers
Source: J Immunother Cancer. 2021 May 10;9(5):e001942. doi: 10.1136/jitc-2020-001942 (PMC8112417; doi:10.1136/jitc-2020-001942)
Supplement: Supplementary data [file jitc-2020-001942supp001.pdf]

Supplementary Methods

Cell-free DNA copy number variations predict efficacy of immune checkpoint inhibitor-based therapy in hepatobiliary cancers

DNA extraction

Whole blood samples were centrifuged in Streck tubes at 1600 x g for 10 minutes at room temperature to separate plasma. The upper plasma layer was removed and transferred to a new 1.5 mL tube, followed by centrifugation of the plasma at 16000 x g for 10 minutes at room temperature. Plasma cell-free DNA (cfDNA) was isolated using the MagMAX Cell-free DNA Isolation Kit (Life Technologies, California, USA) according to the manufacturer's instructions. To isolate genomic DNA (gDNA), we centrifuged whole blood and collected the buffy coat layer to separate the white blood cell samples, and then we used the TIANamp Genomic DNA Kit (Tiangen, Beijing, China) according to the manufacturer's protocol. The concentrations of purified DNA were determined by the Qubit dsDNA HS Assay Kit with the Qubit 4.0 Fluorometer (Life Technologies, California, USA).

Library construction, target capture and next-generation sequencing

The plasma cfDNA and blood cell DNA mutation profiles were assessed by a cancer gene–targeted NGS panel in Genecast Biotechnology Co Ltd China laboratory. The cancer gene-targeted NGS was designed by Genecast and commercially available(Genecast, Wuxi, China). Genomic DNA was sheared into 150-200 bp fragments with a Covaris M220 Focused-ultrasonicator instrument (Covaris, Massachusetts, USA). Fragmented gDNA and cfDNA libraries were constructed by the KAPA Hyper Prep Kit (Kapa Biosystems, Massachusetts, USA) according to the manufacturer's instructions. The concentrations and size distributions of the libraries were analysed by a Qubit 4.0 Fluorometer (Life Technologies, California, USA) and an Agilent Bioanalyzer 2100 (Agilent Technologies, USA). DNA libraries were captured

Yang X, *et al. J Immunother Cancer* 2021; 9:e001942. doi: 10.1136/jitc-2020-001942

following the NimbleGen SeqCap EZ Library SR (Roche, Wisconsin, USA) User's Guide with a designed Genescope panel (Genecast, Wuxi, China) that included major tumour-related genes. The captured libraries were sequenced with the Illumina NovaSeq 6000 platform (Illumina, California, USA) to produce 150 paired-end sequences according to the manufacturer's instructions.

Mapping and single-nucleotide variant (SNV) and copy number variation (CNV) calling

The SNV and CNV calling of cfDNA was according to the standard pipeline in previously published study which also used the Genecast's cancer gene–targeted NGS panel.[1] The paired-end reads generated from the NovaSeq 6000 platform were mapped to the hg19 reference genome (NCBI Build 37.5) with BWA 0.7.17 version (default parameters)[2]. Then, the Picard toolkit (v 2.1.0) and Genome Analysis ToolKit (v 3.7) were used to make duplicates and for realignment.VarDict (v 1.5.1)[3] was used for variant calling to plasma samples and matched white blood cell samples of each patient, while compound heterozygous mutations were merged by FreeBayes (v 1.2.0)[4]. After annotation through ANNOVAR[5], somatic mutations were selected using the following criteria: i) located in intergenic or intronic regions; ii) identified as synonymous SNVs; iii) allele frequency >= 0.002 in the Exome Aggregation Consortium (ExAC)[6] and gnomAD databases; iv) allele frequency <0.01 in plasma samples; v) strand bias mutations in the reads; vi) support reads <5; and vii) depth <30.

For CNV calling, we used 30 health control blood samples to construct copy number baseline as negative control and used software ctCNV to call copy number variation for each case. First, counting the read count for each target region and normalize the read count for all regions of each sample so that the different samples are comparable. Second, applying rolling median to normalize GC-content and length of target regions. After correcting GC content, target region length, and read count, normalized test samples are compared to the baseline, log2ratios are calculated at each region-level first, and then the median of log2ratios of all regions within the same gene range is used to

represent the log2ratio of the gene. To determine the CNV for each gene, in addition to the absolute copy number, we also calculated the gene specificity score (GCS, represents the degree of gene level difference between the test sample and control), with quantitate the instability of copy number compare to the control samples, and added a statistical test filter to determine whether the GCS is significantly different from control samples. Only genes who are statistically significant and absolute copy number exceed a given threshold will be judged to be CNV.

To determine the copy number, we used white blood cell samples as a negative control and used cnvkit (v 0.9.2) software to call the CNVs from the matched plasma samples of patients[7].

Molecular features

The number of somatic nonsynonymous SNVs in all samples were determined to calculate the tumour mutation burden (TMB) with the following rules: i) not splicing or exonic; ii) sequencing depth <100X and allele frequency < 0.05; iii) allele frequency >=0.002 in the ExAC[6] and gnomAD databases; and iv) strand bias mutations in the reads and other rules. The TMB of the samples was calculated by the absolute mutation counts via the following formula:

$$TMB = \frac{Absolute\ mutation\ counts}{Panel\ exonic\ base\ num} \times 1000000$$

TMB was measured in mutations per Mb[8].

The copy number instability (CNI) score was calculated by the sum of the Z-scores computed by the following step. The log2 read counts were converted into Z-scores based on Gaussian transformations versus a normal control group after the correction of GC content and length of target region using proprietary algorithms for each region. If the Z-score was greater than the 95th percentile plus twice the absolute standard deviation of the normal control group, the target regions were retained and summed into the CNI score [9].

Molecular mutation burden (MMB) was calculated by the mean frequency among the obtained mutations for each gene of each patient. Details could be seen in the previous study.[10]

The somatic mutations of ten oncogenic signalling pathways, including the cell cycle, Hippo, Myc, Notch, Nrf2, PI-3-Kinase/Akt, RTK-RAS, TGFβ signalling, p53 and β-catenin/Wnt pathways, were analysed as described in a previous publication.[11]

Supplementary References

1. Jiang T, Jiang L, Dong X, et al. Utilization of circulating cell-free DNA profiling to guide first-line chemotherapy in advanced lung squamous cell carcinoma. *Theranostics* 2021;11(1):257-67. doi: 10.7150/thno.51243 [published Online First: 2021/01/05]
2. Li H, Durbin R. Fast and accurate short read alignment with Burrows-Wheeler transform. *Bioinformatics* 2009;25(14):1754-60. doi: 10.1093/bioinformatics/btp324 [published Online First: 2009/05/20]
3. Lai Z, Markovets A, Ahdesmaki M, et al. VarDict: a novel and versatile variant caller for next-generation sequencing in cancer research. *Nucleic Acids Res* 2016;44(11):e108. doi: 10.1093/nar/gkw227 [published Online First: 2016/04/10]
4. Garrison E, Marth G. Haplotype-based variant detection from short-read sequencing. *arXiv* 2012;preprint arXiv:1207.3907
5. Wang K, Li M, Hakonarson H. ANNOVAR: functional annotation of genetic variants from high-throughput sequencing data. *Nucleic Acids Res* 2010;38(16):e164. doi: 10.1093/nar/gkq603 [published Online First: 2010/07/06]
6. Karczewski KJ, Weisburd B, Thomas B, et al. The ExAC browser: displaying reference data information from over 60 000 exomes. *Nucleic Acids Res* 2017;45(D1):D840-d45. doi: 10.1093/nar/gkw971 [published Online First: 2016/12/03]
7. Talevich E, Shain AH, Botton T, et al. CNVkit: Genome-Wide Copy Number Detection and Visualization from Targeted DNA Sequencing. *PLoS Comp Biol* 2016;12(4):e1004873. doi: 10.1371/journal.pcbi.1004873 [published Online First: 2016/04/23]
8. Chalmers ZR, Connelly CF, Fabrizio D, et al. Analysis of 100,000 human cancer genomes reveals the landscape of tumor mutational burden. *Genome Med* 2017;9(1):34. doi: 10.1186/s13073-017-0424-2 [published Online First: 2017/04/20]
9. Weiss GJ, Beck J, Braun DP, et al. Tumor Cell-Free DNA Copy Number Instability Predicts Therapeutic Response to Immunotherapy. *Clin Cancer Res* 2017;23(17):5074-81. doi: 10.1158/1078-0432.Ccr-17-0231 [published Online First: 2017/03/23]
10. Jiang T, Li X, Wang J, et al. Mutational Landscape of cfDNA Identifies Distinct Molecular Features Associated With Therapeutic Response to First-Line Platinum-Based Doublet Chemotherapy in Patients with Advanced NSCLC. *Theranostics* 2017;7(19):4753-62. doi: 10.7150/thno.21687 [published Online First: 2017/12/01]
11. Sanchez-Vega F, Mina M, Armenia J, et al. Oncogenic Signaling Pathways in The Cancer Genome Atlas. *Cell* 2018;173(2):321-37.e10. doi: 10.1016/j.cell.2018.03.035 [published Online First: 2018/04/07]
