# Supplementary material for: Cell-free DNA copy number variations predict efficacy of immune checkpoint inhibitor-based therapy in hepatobiliary cancers
Source: J Immunother Cancer. 2021 May 10;9(5):e001942. doi: 10.1136/jitc-2020-001942 (PMC8112417; doi:10.1136/jitc-2020-001942)
Supplement: Supplementary data [file jitc-2020-001942supp002.pdf]

**Supplementary Table 1. Other clinical characteristics of patients with hepatobiliary cancer (N = 187)**

| Variable | ICI cohort 1 (N=43) | ICI Cohort2 (N=108) | Non-ICI Cohort (N=36) |
|---|---|---|---|
| Etiology — no. (%) | | | |
| Hepatitis B | 17(39.5) | 46(42.6) | 10(27.8) |
| Hepatitis C | 0(0) | 3(2.8) | 2(5.6) |
| NAFLD | 2(4.7) | 6(5.6) | 1(2.8) |
| AFLD | 3(7.0) | 4(3.7) | 1(2.8) |
| Alcohol use | 3(7.0) | 8(7.4) | 1(2.8) |
| Others | 24(55.8) | 53(49.0) | 23(63.8) |
| Biliary stone — no. (%) | | | |
| Y | 9(20.9) | 27(25.0) | 8(22.2) |
| N | 32(74.4) | 78(72.2) | 24(66.7) |
| NA | 2(4.7) | 3(2.8) | 4(11.1) |
| Macrovascular Invasion — no. (%) | | | |
| Y | 6(14.0) | 26(24.1) | 3(8.3) |
| N | 37(86.0) | 82(75.9) | 33(91.7) |
| TNM Stage — no. (%) | | | |
| I | 0 (37.2) | 2 (1.9) | 4(11.1) |
| II | 6(14.0) | 9(8.3) | 5(13.9) |
| III | 10(23.3) | 32(29.6) | 10(27.8) |
| IV | 27(62.8) | 65(60.2) | 17(47.2) |
| Distant metastasis — no. (%) | | | |
| Y | 23(53.5) | 50(46.3) | 15(41.7) |
| N | 20(46.5) | 58(53.7) | 21(58.3) |
| ALBI score — no. (%) | | | |
| 1 | 30(69.8) | 72(66.7) | 21(58.3) |
| 2 | 13(30.2) | 33(30.6) | 12(33.3) |
| 3 | 0(0.0) | 3(2.8) | 3(8.3) |
| AFP>400ng/ml — no. (%) | | | |
| Y | 3(7.0) | 16(14.8) | 4(11.1) |
| N | 39(90.7) | 88(81.5) | 32(88.9) |
| NA | 1(2.3) | 4(3.7) | 0(0.0) |
| CA19-9>100U/L — no. (%) | | | |
| Y | 18(41.9) | 42(38.9) | 11(30.6) |
| N | 24(55.8) | 63(58.3) | 25(69.4) |
| NA | 1(2.3) | 3(2.8) | 0(0.0) |
| Maximum tumor diameter — no. (%) | | | |
| >=5 cm | 25(58.1) | 54(50.0) | 11(30.6) |

| Variable | ICI cohort 1 (N=43) | ICI Cohort2 (N=108) | Non-ICI Cohort (N=36) |
|---|---|---|---|
| <5 cm | 18(41.9) | 54(50.0) | 25(69.4) |
| Histological Grade — no. (%) | | | |
| Poorly differentiated（G3/G4） | 14(32.6) | 40(37.0) | 9(25.0) |
| Moderately or well differentiated (G1/G2) | 13(30.2) | 34(31.5) | 12(33.3) |
| NA | 16(37.2) | 34(31.5) | 15(41.7) |
| PD-L1 expression — no. (%) | | | |
| TPS >=1% | 4(9.3) | 15(13.9) | 3(8.3) |
| TPS<1% | 22(51.2) | 39(36.1) | 17(47.2) |
| NA | 17(39.5) | 54(50.0) | 16(44.4) |
| Best response rate — no. (%) | | | |
| PR | 6(14.0) | 18(16.7) | 3(8.3) |
| SD | 18(41.9) | 62(57.4) | 14(38.9) |
| PD | 16(37.1) | 24(22.2) | 3(8.3) |
| NA | 3(7.0) | 4(3.7) | 16(44.4) |
| Clinical benefit — no. (%) | | | |
| DCB | 13(30.2) | 54(50.0) | 11(30.6) |
| NCB | 30(69.8) | 51(47.2) | 10(27.8) |
| NA | 0(0.0) | 3(2.8) | 15(41.7) |
| PFS — Median (95%CI) month | | | |
| PFS-All | 4.16(2.84-5.47) | 4.13(2.14-6.13) | 6.03(3.87-8.17) |
| PFS-HCC | 10.73(1.62-19.84) | 6.63(3.77-9.49) | 6.03(3.53-8.54) |
| PFS-BTC | 3.06(1.26-4.88) | 3.5(3.00-4.01) | 4.93(3.54-6.32) |
| PFS-CHCC | 2.66(2.51-2.83) | NA | NA |
| OS — Median (95%CI) month | | | |
| OS-All | 13.53(8.10-19.0) | 12.73(9.23-16.24) | 10.07(6.96-13.17) |
| OS-HCC | NR | 17.43(8.06-26.81) | 8.33(7.79-8.88) |
| OS -BTC | 10.13(6.56-13.71) | 12.03(8.78-15.28) | 9.79(3.51-41.96) |
| OS -CHCC | NA | NA | NA |

Abbreviations: OS: overall survival, PFS: progression-free survival, CI: confidence interval, PR: partial response, SD: stable disease, PD: progressive disease, DCB: durable clinical benefit, NCB: no clinical benefit, NA: not available ,NR: not reach, NAFLD: Non-alcoholic fatty liver disease, AFLD: alcoholic fatty liver disease
