# Supplementary figures and images for: Cell-free DNA copy number variations predict efficacy of immune checkpoint inhibitor-based therapy in hepatobiliary cancers
Source: J Immunother Cancer. 2021 May 10;9(5):e001942. doi: 10.1136/jitc-2020-001942 (PMC8112417; doi:10.1136/jitc-2020-001942)

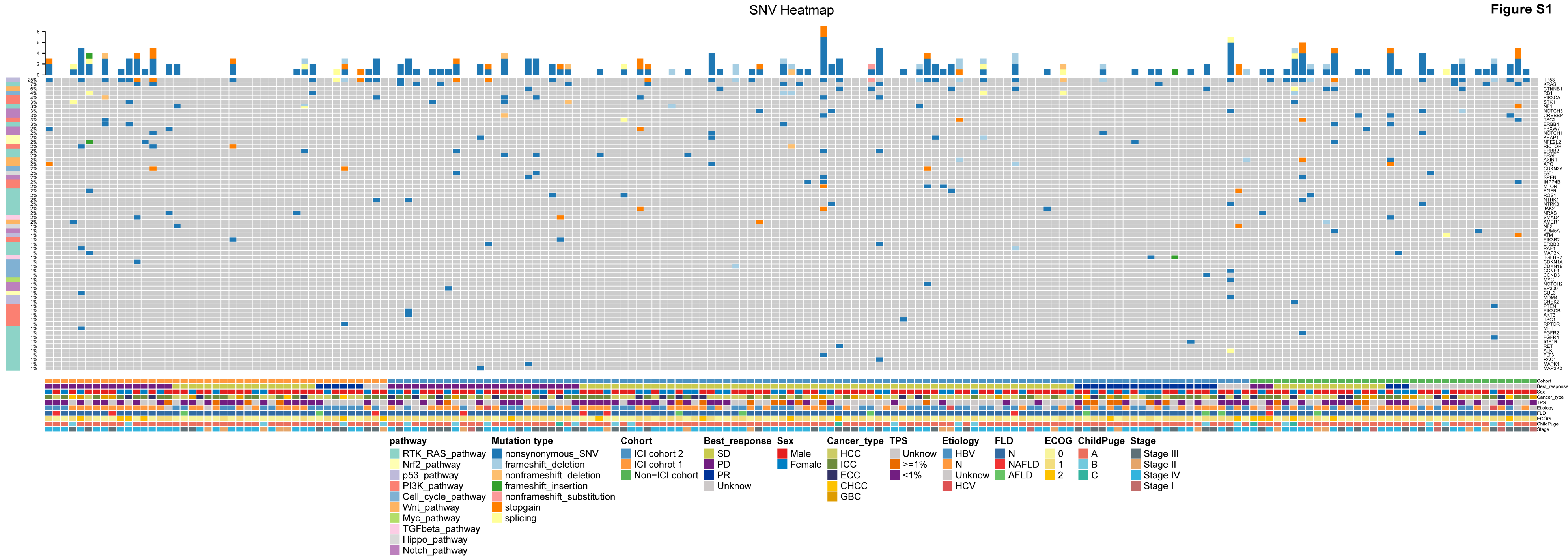

Supplement: Supplementary data [file jitc-2020-001942supp003.pdf]

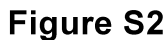

Supplement: Supplementary data [file jitc-2020-001942supp004.pdf]

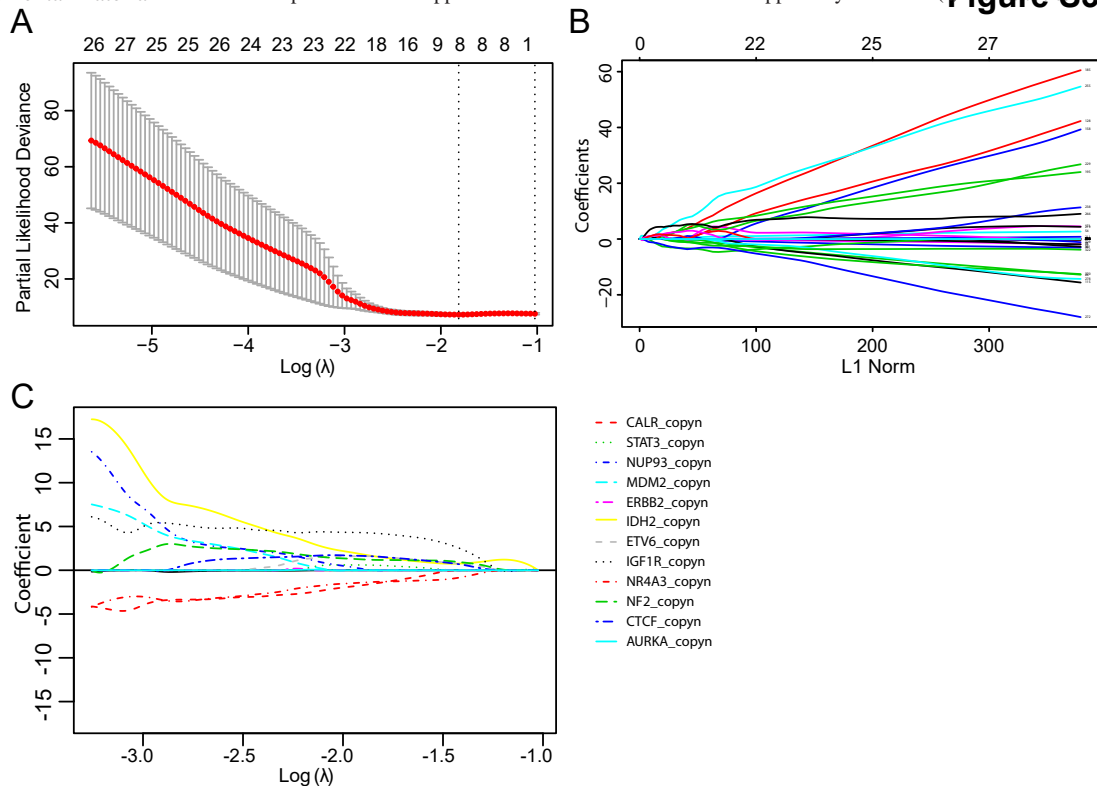

Supplement: Supplementary data [file jitc-2020-001942supp005.pdf]

# **A** **Figure S5**

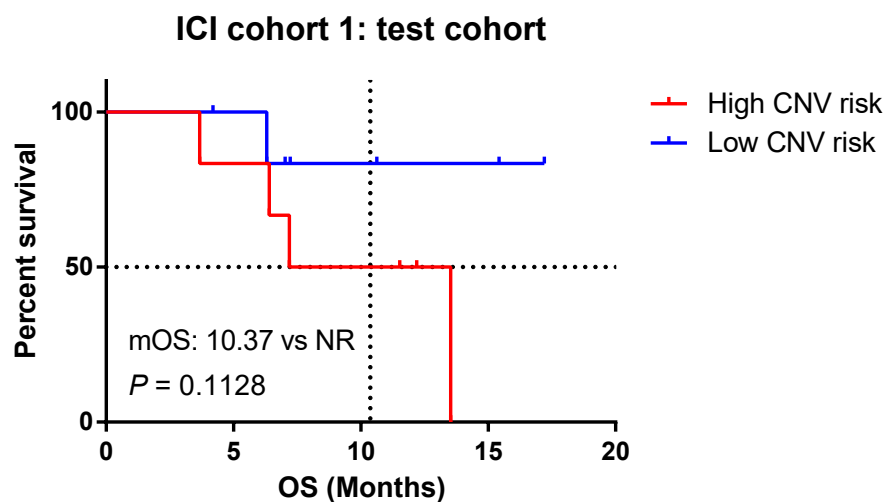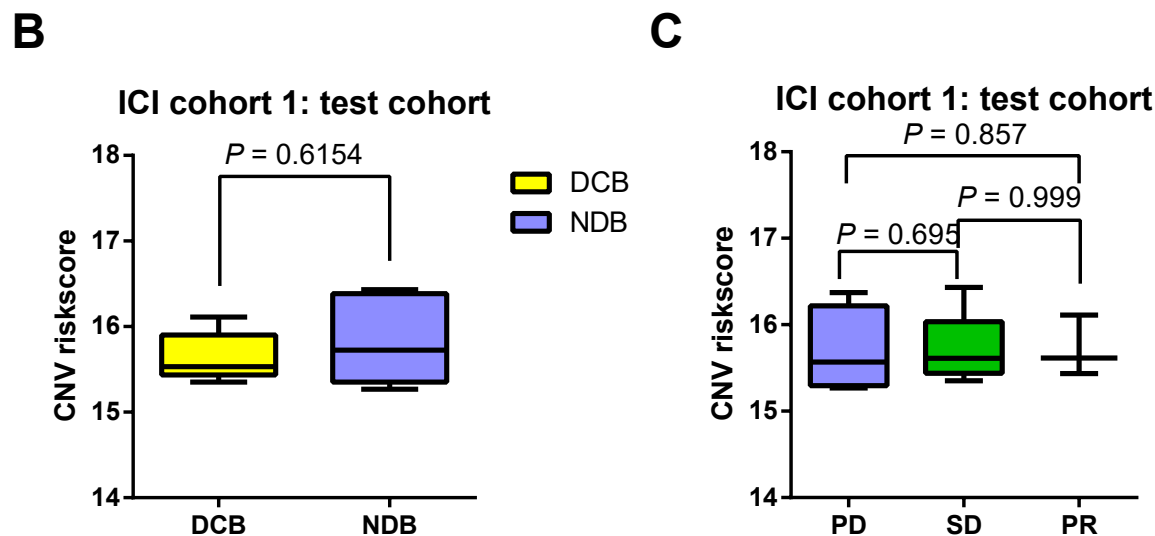

Supplement: Supplementary data [file jitc-2020-001942supp006.pdf]

**Figure S4**

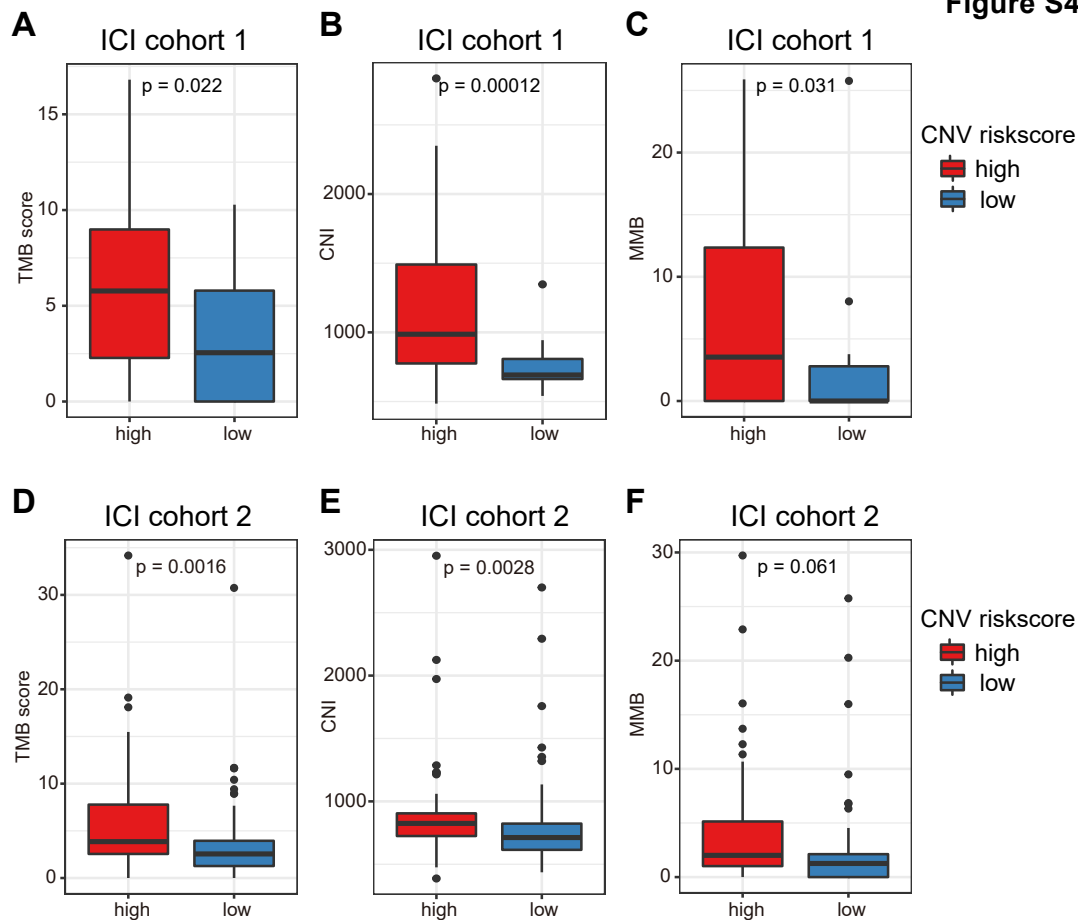

Supplement: Supplementary data [file jitc-2020-001942supp008.pdf]

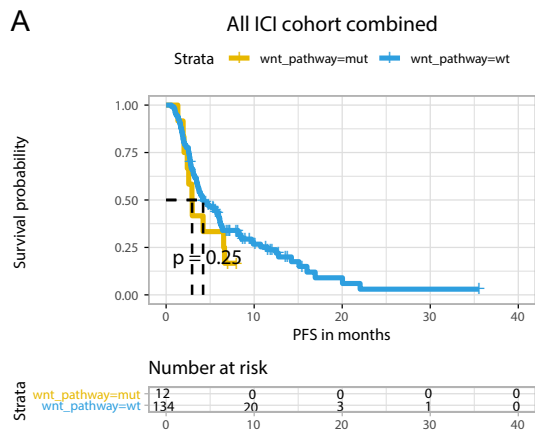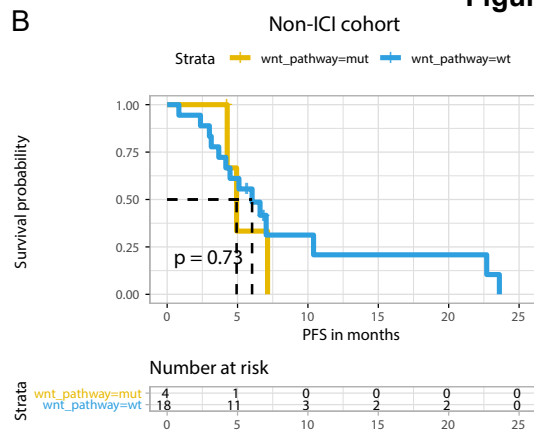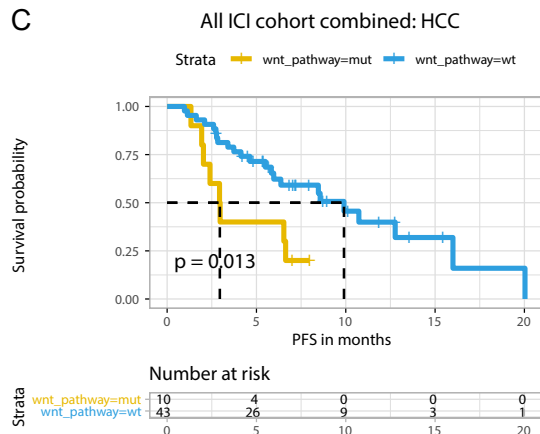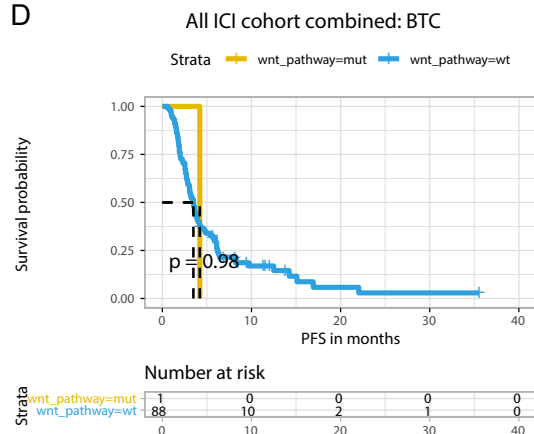

Supplement: Supplementary data [file jitc-2020-001942supp009.pdf]

Figure R2

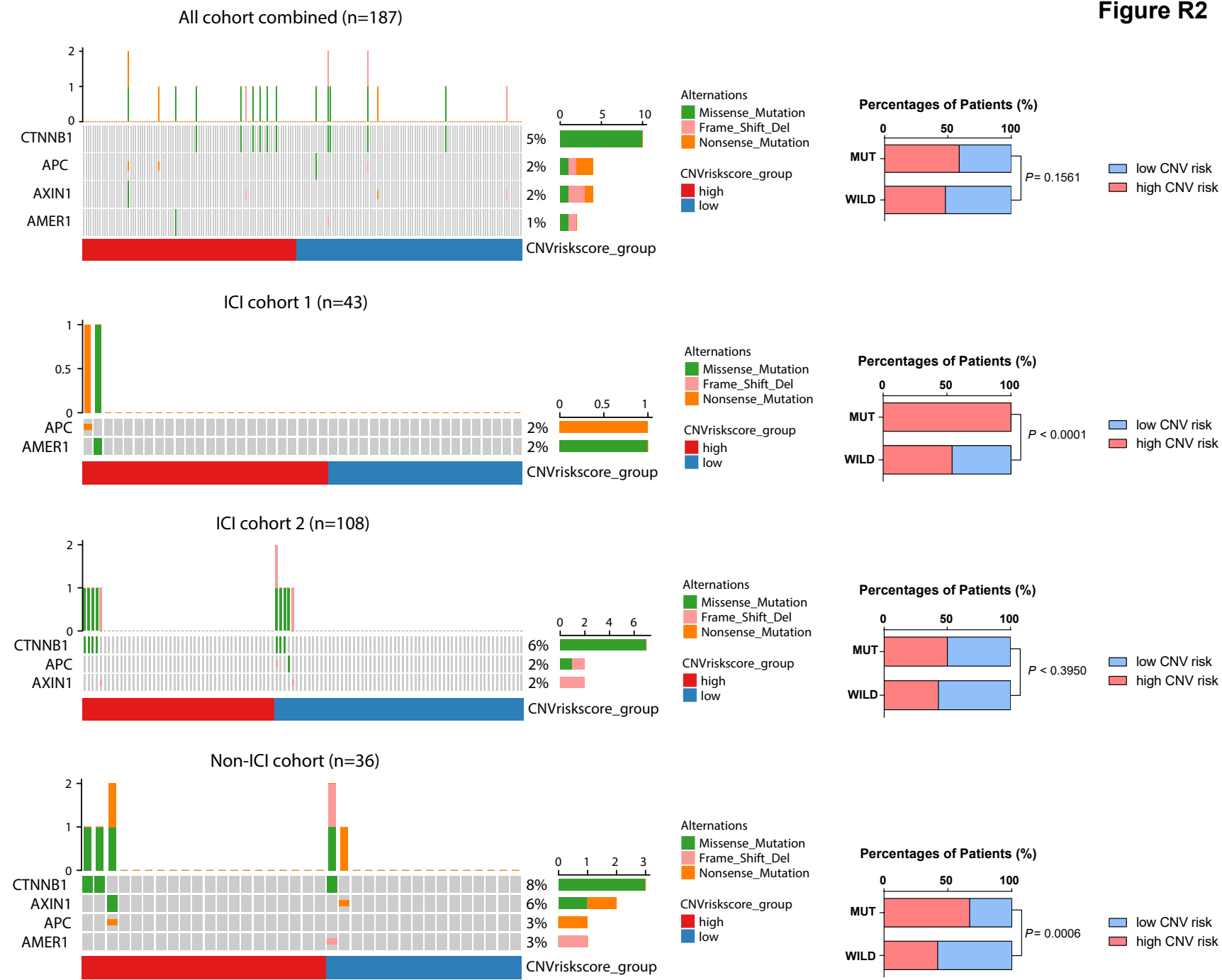

Supplement: Supplementary data [file jitc-2020-001942supp010.pdf]
