# Supplementary material for: Cell-free DNA copy number variations predict efficacy of immune checkpoint inhibitor-based therapy in hepatobiliary cancers
Source: J Immunother Cancer. 2021 May 10;9(5):e001942. doi: 10.1136/jitc-2020-001942 (PMC8112417; doi:10.1136/jitc-2020-001942)
Supplement: Supplementary data [file jitc-2020-001942supp007.pdf]

Supplementary Table 2. The correction between CNV risk score with clinicopathological characteristics

Variable	Category	High-risk (N=71)	Low-risk (N=80)	P value (Fisher's exact test)
**Sex**	Female	28 (39.4)	25 (47.2)	0.310
	Male	43 (60.6)	55 (56.1)	
**Age**	<60	34 (47.9)	39 (48.8)	1
	>=60	37 (52.1)	41 (51.2)	
**Histological Type**	BTC	52 (73.2)	39 (48.8)	**0.004**
	CHCC	2 (2.8)	2 (2.6)	
	HCC	17 (23.9)	39 (48.8)	
**Histological Grade**	Moderately or well differentiated	42 (59.2)	55 (68.8)	0.238
	Poorly differentiated	29 (40.8)	25 (31.3)	
**Macrovascular Invasion**	N	54 (76.1)	65 (81.3)	0.550
	Y	17 (23.9)	15 (18.8)	
**TNM Stage**	I-III	30 (42.3)	29 (36.3)	0.505
	IV	41 (57.7)	51 (63.7)	
**Tumor burden score**	<8	37 (52.1)	57 (71.3)	**0.019**
	>=8	34 (47.9)	23 (28.7)	
**Maximum tumor diameter**	<5	27 (38.0)	52 (65.0)	**0.001**
	>=5	44 (62.0)	28 (35.0)	

Abbreviations: HCC: hepatocellular carcinoma, BTC: biliary tract cancer, CHCC: combined hepatocellular-cholangiocarcinoma, CNV: copy number variation
