# Supplementary material for: Cell-free DNA copy number variations predict efficacy of immune checkpoint inhibitor-based therapy in hepatobiliary cancers
Source: J Immunother Cancer. 2021 May 10;9(5):e001942. doi: 10.1136/jitc-2020-001942 (PMC8112417; doi:10.1136/jitc-2020-001942)
Supplement: Supplementary data [file jitc-2020-001942supp011.pdf]

北京協和醫院
PEKING UNION MEDICAL COLLEGE HOSPITAL

PROTOCOL NUMBER: [JS-1864]

PROTOCOL TITLE: [Lenvatinib Combined Pembrolizumab in Advanced Hepatobiliary Tumors]

IRB APPROVAL DATE: **[2019-01-22]**

IRB APPROVAL TYPE: **Standard Review**

Dear Dr. Haitao Zhao

The Institutional Review Board (IRB) of Peking Union Medical College Hospital (PUMCH)has reviewed the above-named protocol and has determined that 1) the applicant(s) are qualified to conduct the proposed study; 2) the design of the study is rationale and scientifically sound; 3) human subject protection is adequate and 4) the informed consent is acceptable. The IRB thus approve the protocol.

This approval is valid for one year from the date of IRB review and will no longer be in effect on the date listed above as the IRB expiration date. A Continuing Review application must be approved before the expiration date to avoid cessation of all research activities.

It is the responsibility of all investigators and research staff to promptly report to the IRB any adverse and unexpected events, protocol deviation or violations and unanticipated problems which may impose risks on subjects.

Signature of Chairman of IRB member: Zhaohui Zhu

PUMCH Institutional Review Board
